# The 2024 UK asthma guidelines: considerations for patients with asthma not responding to maintenance and reliever therapy

**DOI:** 10.3399/BJGP.2025.0629

**Published:** 2026-05-01

**Authors:** Liam G Heaney, Dominick Shaw, Paul Pfeffer, Ian Pavord

**Affiliations:** 1 Wellcome-Wolfson Institute for Experimental Medicine, School of Medicine, Dentistry and Biomedical Sciences, Queen’s University Belfast, Belfast, UK; 2 NIHR Leicester BRC, Department of Respiratory Sciences, University of Leicester, Leicester, UK; 3 Department of Respiratory Medicine, Barts Health NHS Trust, London, UK; 4 Oxford University, Nuffield department of Medicine, Respiratory Medicine, Oxford, UK

## Introduction

In 2024, the British Thoracic Society (BTS), National Institute for Health and Care Excellence (NICE), and Scottish Intercollegiate Guidelines Network (SIGN) published updated guidelines for asthma diagnosis, monitoring, and management in the UK (Figure 1).^1^ This new clinical asthma pathway departs from previous guidelines, aligning more closely with the preferred (track 1) Global Initiative for Asthma (GINA) recommendations.^2^ The 2024 BTS/NICE/SIGN NG245 guidelines (hereinafter referred to as the 2024 UK asthma guidelines) are a definite improvement from the last iteration, following a stepwise approach that considers asthma as a disease of airway inflammation, based on evidence of the benefits of steroid-containing combination inhalers.^1^ The guidelines aim to offer clarity for asthma diagnosis and enhance asthma control at the population level by reducing over-reliance on short-acting β_2_-agonists (SABAs) and increasing use of as-needed anti-inflammatory reliever therapy, notably inhaled corticosteroids (ICSs) combined with rapid-onset β_2_-agonists.^1^ Nonetheless, there remains uncertainty on how to manage individuals who experience exacerbations and troublesome symptoms despite having stepped up to moderate-dose maintenance and reliever therapy (MART) (Figure 1).

**Figure 1.**
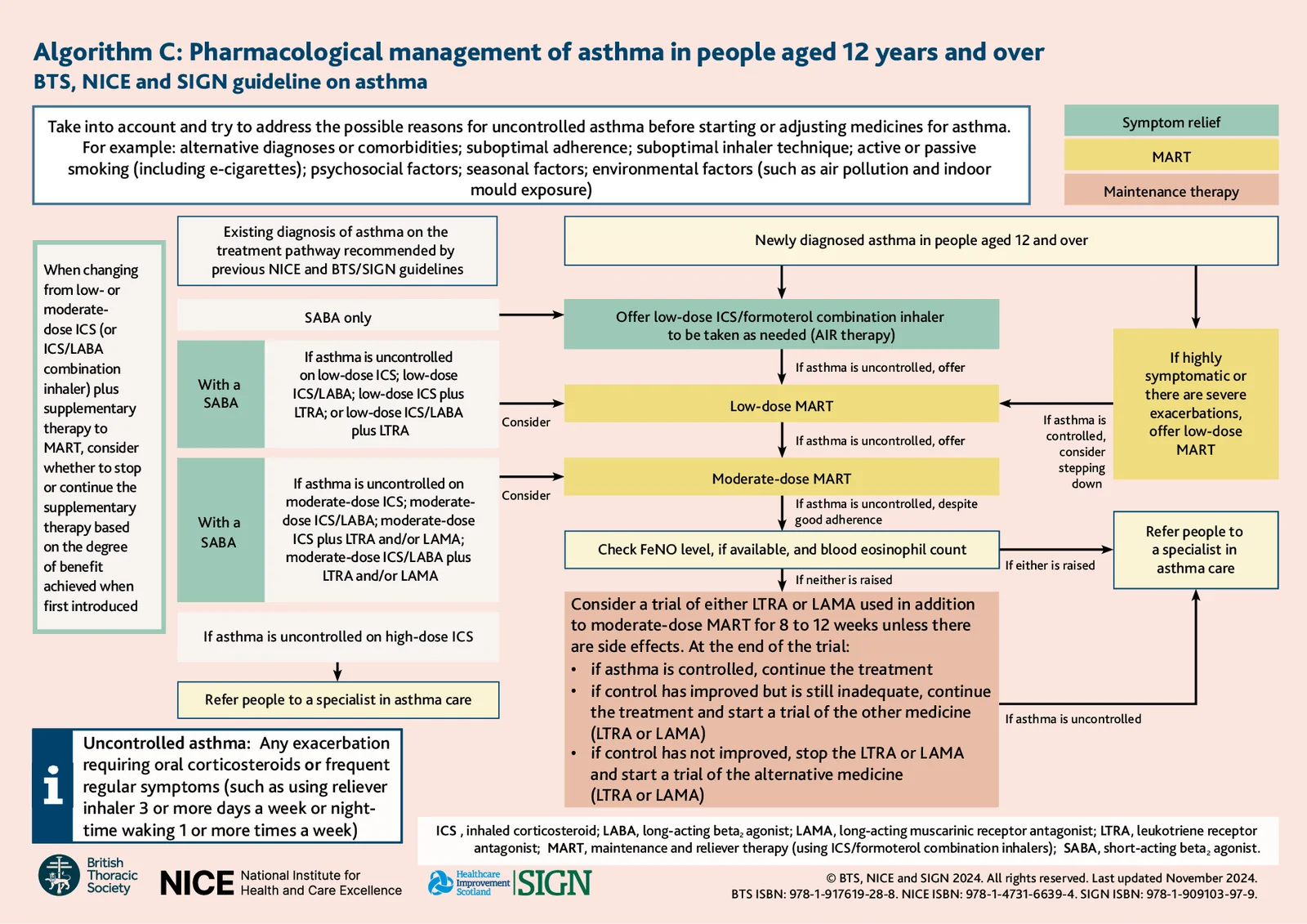
BTS/NICE/SIGN NG245 guidelines on asthma. © NICE [2024] ALGORITHM C: PHARMACOLOGICAL MANAGEMENT OF ASTHMA IN PEOPLE AGED 12 YEARS AND OVER BTS, NICE, AND SIGN GUIDELINE ON ASTHMA. All rights reserved. subject to notice of rights. Available from https://www.nice.org.uk/guidance/ng245/resources/algorithm-c-pharmacological-management-of-asthma-in-people-aged-12-years-and-over-bts-nice-pdf-13556516367.

The 2024 UK asthma guidelines call for initial assessment of type-2 (T2) airway inflammation biomarkers;^1^ this is important as, for the first time, disease activity will be assessed in the early stages of asthma, offering the prospect of prompt intervention targeted at airway inflammation, better recognition of high risk disease and potential disease modification. However, the guidelines recommend symptom-driven maintenance therapy and are more equivocal about the role of biomarkers in stepping patients between anti-inflammatory reliever-only therapy and different doses of MART. This leads to concerns about the appropriateness of symptom-directed use of MART; particularly overuse in low-risk patients with high symptoms but low biomarkers of T2 inflammation, or underuse in high-risk patients with low symptoms but high T2 inflammation.^3^

Here, a panel of UK asthma specialists aim to supplement these 2024 UK asthma guidelines to provide a practical approach to managing patients at the individual level regarding key areas where healthcare professionals may benefit from additional guidance. We argue that early assessment of T2 airway inflammation allows maintenance therapy to be biomarker-guided and suggest that this will lead to better long-term outcomes.^3^

## Perspectives from specialists in asthma care

### Key considerations for the place of SABAs and ICS/LABAs in asthma management

The 2024 UK asthma guidelines recommend that patients with newly diagnosed asthma are initiated on low-dose ICS/formoterol (FOR) as-needed, which may be stepped-up to MART.^1^ Single-inhaler MART can be used up to eight puffs per day for budesonide/FOR, and more frequently during an exacerbation.^4^ Patients receiving moderate-dose MART who still experience exacerbations should be considered for add-on leukotriene receptor antagonist (LTRA) or long-acting muscarinic antagonist (LAMA) therapy (Figure 1). An advantage of the updated guidelines is that when patients are failing moderate-dose MART, repeat assessment of T2 biomarkers is recommended;^1^ this will identify high-risk patients with persisting inflammation who should be referred to a specialist, and stop the symptom-driven escalation of corticosteroid treatment in ‘biomarker low’ patients, which is unlikely to be effective.^3^

Use of single-inhaler MART may be more convenient to patients than using separate maintenance and reliever inhalers,^4^ yet some patients may prefer a once-daily maintenance inhaler;^5,6^ research is needed to compare efficacy of a once-daily ICS/long-acting β_2_-agonists (LABA) with ICS/SABA or SABA as a reliever versus MART. Furthermore, studies are needed to compare beclomethasone/FOR and budesonide/FOR MART inhalers to other combinations of ICS with a rapid-onset bronchodilator.

### Role of biomarkers in asthma management as detailed in the 2024 UK asthma guidelines

Cluster analyses have shown that asthma symptoms do not always correlate with the degree of T2 airway inflammation.^3^ Physicians often focus on suppressing symptoms rather than exacerbations;^7^ however for effective management of asthma, underlying airway inflammation and exacerbation risk must be considered.^3,8^ The 2024 UK asthma guidelines introduce T2 biomarker measurement to routine asthma care and address the dangers of SABA over-reliance.^1^ High levels of blood eosinophils or fractional exhaled nitric oxide (FeNO) are associated with a higher risk of exacerbations and a better response to treatment targeting T2 inflammation for example, corticosteroids; evaluation of these biomarkers is therefore critical for deciding on appropriate maintenance treatment to achieve asthma control.^9^ Combined low-dose ICS with a rapid-onset β_2_-agonist (such as the rapid onset LABA combination of ICS/FOR, and in some other countries [excluding the UK] ICS/SABA) should be prescribed to all patients who present with asthma at diagnosis (Figure 1).^1,2^ We have previously suggested that if asthma control does not improve, a combined approach assessing symptoms and biomarkers of T2 inflammation should guide treatment decisions;^3^ identifying patients who, despite treatment with high-dose ICS/LABA, have T2 airway inflammation and may be candidates for earlier progression to biologic therapy.^3^ Furthermore, if biomarkers remain elevated despite well-controlled symptoms, stepping up to higher dose maintenance ICS/LABA or add-on LAMA or LTRA may still reduce future exacerbation risk.^10^ Importantly, symptom-directed MART can be associated with less complete suppression of T2 inflammation compared to ICS treatment,^3^ and patients with high biomarkers despite low symptoms may be at particularly high risk of persistent T2 inflammation, airway remodelling and sudden, life-threatening exacerbations since prolonged periods without ICS treatment are possible.^11^ ‘Symptom low, inflammation high, risk high’ patients are at particular danger of remaining on low-dose ICS/FOR as-needed, given the absence of regular symptoms that would prompt escalation to MART, leading to ongoing airway inflammation and subsequent development of severe exacerbations and/or long-term airway remodelling. A personalised ‘treatable traits’ approach, which has largely been advocated for specialist care and patients with complex airways disease, should ideally be used to guide treatment decisions in primary care.^8^

Future clinical studies are needed to access whether biomarkers should be measured in primary care on an annual basis for all patients with asthma.^3^ This could guide optimal ICS/LABA dosing to reduce future risk of exacerbations and loss of lung function. Similar to other chronic conditions, asthma management should involve repeated measurements to reduce exacerbation risk rather than only treating immediate symptoms.^12^ Poor access to FeNO testing in primary care has been identified as a barrier and requires investment.^3^

### Identifying when GPs should refer patients to specialists

The 2024 UK asthma guidelines state that referral to specialists should be made when FeNO and/or blood eosinophil count is raised or when asthma is not controlled despite treatment with moderate-dose MART, LTRA, LAMA or high-dose ICS.^1^ It is unclear how to define ‘failure’ of MART therapy and how it could be measured, in other words, is counting inhalers the ideal metric and how many inhalers per year would be considered MART ‘failure’? Future studies are needed to define MART failure and determine how many patients overuse or underuse MART. Furthermore, guidance is needed on when patients should be referred to a specialist or which therapies should be trialled prior to starting a biologic. In addition, a study is needed to assess the number of patients referred to specialists in the UK and how their asthma is managed to better describe the provision needed by the healthcare service. Importantly, there are delays between referral to specialist care and assessment in clinic; guidelines need to be cognisant of this, for example, by suggesting patients with high T2 inflammatory biomarkers failing on moderate-dose MART should be tried on once-daily high-dose ICS/LABA while waiting for an appointment in specialist care, acknowledging that adherence to maintenance doses is often low in asthma but improves in many patients after switching to once-daily maintenance inhalers. Further research is also needed to identify which patients respond best to the addition of a LAMA, rather than simply selecting patients based on the absence of high T2 biomarkers, and how best to add a LAMA to MART regimens.

## Conclusions

By acknowledging asthma as a disease of airway inflammation, the 2024 UK asthma guidelines are a definite improvement and are appropriate at a population level; nonetheless questions persist around treating patients who still experience exacerbations despite having stepped up to moderate-dose MART. Implementation of the guidelines in clinical practice over the next 3–5 years provides an opportunity to shift to a biomarker-directed maintenance treatment approach for the next iteration.
